# Deep Sequencing Reveals Novel Genetic Variants in Children with Acute Liver Failure and Tissue Evidence of Impaired Energy Metabolism

**DOI:** 10.1371/journal.pone.0156738

**Published:** 2016-08-02

**Authors:** C. Alexander Valencia, Xinjian Wang, Jin Wang, Anna Peters, Julia R. Simmons, Molly C. Moran, Abhinav Mathur, Ammar Husami, Yaping Qian, Rachel Sheridan, Kevin E. Bove, David Witte, Taosheng Huang, Alexander G. Miethke

**Affiliations:** 1 Divisions of Human Genetics, Cincinnati Children’s Hospital Medical Center, 3333 Burnet Avenue, Cincinnati, OH 45229–3039, United States of America; 2 Pediatric Gastroenterology, Hepatology and Nutrition, Cincinnati Children’s Hospital Medical Center, 3333 Burnet Avenue, Cincinnati, OH 45229–3039, United States of America; 3 Pediatric Pathology, Cincinnati Children’s Hospital Medical Center, 3333 Burnet Avenue, Cincinnati, OH 45229–3039, United States of America; 4 Department of Pediatrics, University of Cincinnati College of Medicine, Cincinnati, Ohio, United States of America; Instituto de Investigación Hospital 12 de Octubre, SPAIN

## Abstract

**Background & Aims:**

The etiology of acute liver failure (ALF) remains elusive in almost half of affected children. We hypothesized that inherited mitochondrial and fatty acid oxidation disorders were occult etiological factors in patients with idiopathic ALF and impaired energy metabolism.

**Methods:**

Twelve patients with elevated blood molar lactate/pyruvate ratio and indeterminate etiology were selected from a retrospective cohort of 74 subjects with ALF because their fixed and frozen liver samples were available for histological, ultrastructural, molecular and biochemical analysis.

**Results:**

A customized next-generation sequencing panel for 26 genes associated with mitochondrial and fatty acid oxidation defects revealed mutations and sequence variants in five subjects. Variants involved the genes *ACAD9*, *POLG*, *POLG2*, *DGUOK*, and *RRM2B*; the latter not previously reported in subjects with ALF. The explanted livers of the patients with heterozygous, truncating insertion mutations in *RRM2B* showed patchy micro- and macrovesicular steatosis, decreased mitochondrial DNA (mtDNA) content <30% of controls, and reduced respiratory chain complex activity; both patients had good post-transplant outcome. One infant with severe lactic acidosis was found to carry two heterozygous variants in *ACAD9*, which was associated with isolated complex I deficiency and diffuse hypergranular hepatocytes. The two subjects with heterozygous variants of unknown clinical significance in *POLG* and *DGUOK* developed ALF following drug exposure. Their hepatocytes displayed abnormal mitochondria by electron microscopy.

**Conclusion:**

Targeted next generation sequencing and correlation with histological, ultrastructural and functional studies on liver tissue in children with elevated lactate/pyruvate ratio expand the spectrum of genes associated with pediatric ALF.

## Introduction

The etiology of rapidly evolving hepatic dysfunction and coagulopathy in children without known chronic liver diseases remains elusive in almost half of them, including in infants [1]. This hampers the ability to institute targeted therapy. Inborn errors of lipid metabolism are well-known causes of ALF and include respiratory chain (RC) disorders and fatty acid oxidation (FAO) defects causing energy deprivation of hepatocytes from impaired oxidative phosphorylation (OXPHOS) or fatty acid beta-oxidation, respectively [2]. In the OXPHOS pathway, a series of redox reactions is linked to electron transfer along the respiratory complex chain localized at the inner mitochondrial membrane. Mitochondrial DNA (mtDNA) encodes 14 subunits of four of these complexes (I, III, IV, and V). Maintenance and integrity of the mitochondrial genome depends on mtDNA replication, repair and replenishment of the intramitochondrial deoxynucleotide stores [3]. Proteins of the mitochondrial replisome are encoded by nuclear genes, namely, *POLG1 and DGUOK*. Autosomal recessive mutations in these genes lead to mtDNA depletion syndrome (MDS). MDS may present in early childhood as hepatocerebral, myopathic or encephalomyopathic disorders, depending on the tissues affected by the reduction of mtDNA copy number [4]. In the hepatocerebral form, hepatic mtDNA depletion causes decreased activity of those complexes containing mtDNA-encoded subunits, subsequent energy deprivation and death of hepatocytes. Compromised RC function leads to accumulation of pyruvate, the end product of anaerobic glycolysis, in the cytosol, where it is converted to lactate by lactate dehydrogenase, accounting for the elevated lactate/pyruvate (L/P) ratio found in the blood of children with MDS [5,6]. Although the plasma L/P ratio is used in clinical practice to screen for mitochondrial hepatopathies, especially in the context of ALF, its sensitivity and specificity in detecting MDS as cause for ALF has not been prospectively evaluated.

Recent studies showed that next-generation sequencing (NGS) was able to detect pathogenic mutations in genes associated with mitochondrial disorders in a number of infants with clinical and biochemical evidence of impaired OXPHOS and in patients with idiopathic ALF [7,8]. Here, we apply this technology to a cohort of patients with idiopathic ALF and elevated blood molar L/P ratio to search for etiological factors unrecognized by comprehensive clinical testing. Despite several clinical clues, and biochemical and imaging results suggesting OXPHOS disorders, establishing the underlying cause with certainty in the context of ALF is challenging [9]. This is of critical importance, as liver transplantation (LTx) for mitochondrial disorders is controversial because of concerns of progression to extra-hepatic multisystem disease and reports of poor outcomes [10].

We sought genotype-phenotype correlations in a cohort of children with indeterminate ALF and elevated blood L/P ratio by performing targeted NGS and analysis of liver histology, mitochondrial ultrastructure and function.

## Patients and Methods

### Patient cohort

The study protocol was approved *a priori* by the Institutional Review Board (IRB) of Cincinnati Children’s Hospital Medical Center (CCHMC) in accordance with the ethical guidelines of the 1975 Declaration of Helsinki. The need to obtain informed consent was waived by the IRB for this retrospective study. Patient samples were de-identified and analyzed anonymously. Medical records of 74 children who presented to CCHMC between January 2000 and December 2012 with ALF, defined as coagulopathy with an INR >1.9 within the first week of disease onset and no evidence of underlying chronic liver disease, were reviewed. Clinically indicated diagnostic testing did not reveal a cause for the ALF in 40 (54%) of the subjects **(Table 1**), based on review of progress notes, discharge summaries, outpatient clinic notes. Results of testing for auto-antibodies (ANA, SMA, LKM), viral serology/ quantitative blood PCR for CMV/EBV, and metabolic evaluation (urine ketones) were available for review in 75, 73 and 80% of the 40 subjects with idiopathic ALF, respectively, indicating comprehensive, yet not complete, evaluation of this cohort. Specific causes for ALF in 34 subjects included acetaminophen ingestion, shock, or viral infection. In comparison, those with ALF of indeterminate etiology were significantly younger and had worse outcomes. Of the 40 subjects with idiopathic ALF, twelve were selected for genotype-phenotype correlation because their blood molar L/P ratios were elevated (≥20) and their frozen liver samples were available for molecular and biochemical studies. Archived samples from non-neoplastic background liver tissue of 12 subjects (ages <5 [n = 4], 5–10 [n = 4] and >10 years [n = 4]) who underwent liver resection for neoplasms were obtained from the CCHMC BioBank. These samples served as age-matched controls for complex activity assays and mtDNA copy number quantitation.

**Table 1:** Clinical characteristics of study subjects with acute liver failure.

	Total number of subjects with ALF from 2000–2012: N = 74	
Etiology	Indeterminate	Identified cause
Number (% of total)	N = 40 (54)	N = 34 (46)
Diagnosis (number of	NA	Autoimmune hepatitis or HLH (12)
patients)		Acetaminophen (8)
		Viral infection (6)
		Wilson’s disease (4)
		Shock (2)
		Mitochondrial disorder (1)
		Cystic fibrosis (1)
Median age at diagnosis in months	50.5 (p = 0.03)*	137
Survival with native liver at 6 months	15 (37.5%) (p = 0.03)**	23 (67.6%)

Statistical tests

* unpaired t test

** Fisher exact test

### NGS, variant interpretation, and comparative genomic hybridization

We developed a HaloPlex enrichment platform for use with Illumina HiSeq 2500 sequencing customized to identify variants in 26 nuclear genes **(Table 2,** including references for genes) belonging to RC and FAO disorders [11]. The gene panel consists of 15 nuclear genes involved in maintenance of mtDNA integrity and in MDS, five genes encoding assembly and translation factors for RC complexes, and six genes associated with FAO defects. Mutations in genes from these three groups have previously been associated with ALF, especially in infants [4,5]. For the DNA enrichment and library preparation, the workflow had 4 major steps; 1) DNA digestion and denaturation, 2) hybridization to probe library, 3) capture and target ligation and 4) PCR amplification of targeted fragments. In short, a total of 0.5 μg of genomic DNA was digested with restriction enzymes. After digestion, eight digestion reactions were pooled, resulting in a single DNA sample containing a 16-enzyme restriction fragment library that included both target and non-target gDNA regions. The restriction fragments were hybridized to the HaloPlex probe capture library, designed to hybridize selectively to target fragments and to direct circularization of such targeted DNA fragments, during which Illumina sequencing motifs were incorporated. Then, the biotinylated-probe/fragment hybrids were retrieved with magnetic streptavidin beads. DNA ligase was added to the capture reaction to catalyze the formation of closed-circular DNA from the circularized target DNA-HaloPlex probe hybrids. Non-target DNA in the capture reaction liquid phase remained in linear fragment form and was digested by the exonuclease activity of the provided Haloase A and B enzymes. During the Haloase B treatment, circularized target DNA was released from the streptavidin beads into the liquid phase. Then, the captured DNA was amplified and index-tagged in PCR reactions containing Illumina Primer 1.0 along with the appropriate index primer followed by a DNA purification using AMPure XP beads. The PCR products from each library was checked by the Agilent Bioanalyzer and quantitated the Qubit® 1.0 Fluorometer (Invitrogen), respectively. The samples with different indexes were pooled at equal molar concentration and sequenced as single-end reads on the HiSeq 2500 (Illumina) instrument according to the manufacturer’s protocols at the DNA sequencing core at Cincinnati Children’s Hospital Medical Center (CCHMC).

**Table 2:** Genes included in customized NGS panel.

Gene (Chromosome)	Exons	Protein	Syndrome
**A. Respiratory Chain (RC) defect disorders**			
**A. 1.a. Mitochondrial DNA Depletion- hepatocerebral form**			
*DGUOK* (2p13.1)	8	DEOXYGUANOSINE KINASE	MDS 3 [3]
*MPV17* (2p23.3)	12	MPV17	MDS 6 [4]
*POLG* (15q26.1)	23	POLYMERASE, DNA, GAMMA	MDS 4a (Alpers type)
			MDS 4b (MNGIE type)
			PEO [5–7]
*C10orf2* "TWINKLE" (10q24.31)	5	CHROMOSOME 10 OPEN READING FRAME 2	MDS 7, PEO [7]
**A. 1.b. Mitochondrial DNA Depletion- encephalomyopathic form**			
*POLG2* (17q23.3)	8	POLYMERASE, DNA, GAMMA-2	PEO [8]
*OPA1* (3q29)	32	DYNAMIN-LIKE 120-KD PROTEIN	Optic atrophy [9]
*OPA3* (19q13.32)	3	OPA3	Optic atrophy 3, MGA3 [10]
*RRM2B* (8q22.3)	10	p53-INDUCIBLE AND RIBONUCLEOTIDE	MDS 8a (renal tubulopathy)
		REDUCTASE SMALL SUBUNIT 2-LIKE	MDS 8b (MNGIE)
			PEO [11]
*SLC25A4* "ANT1" (4q35.1)	4	SOLUTE CARRIER FAMILY 25 (MITOCHONDRIAL CARRIER, ADENINE NUCLEOTIDE TRANSLOCATOR), MEMBER 4	Cardiomyopathy, PEO [12]
*SUCLA2* (13q14.2)	11	SUCCINATE-CoA LIGASE, BETA SUBUNIT	MDS 5 [13]
*SUCLG1* (2p11.2)	10	SUCCINATE-CoA LIGASE, ALPHA SUBUNIT	MDS 9 [13]
*SUCLG2* (3p14.3)	13	SUCCINATE-CoA LIGASE, BETA SUBUNIT	MDS [14]
**A. 1.c. Mitochondrial DNA Depletion- myopathic form**			
*TK2* (16q21)	13	THYMIDINE KINASE, MITOCHONDRIAL	MDS 2 [15]
*TYMP* (22q13.33)	9	THYMIDINE PHOSPHORYLASE	MDS 1 [16]
			PEO
*MGME1* (20p11.23)	4	CHROMOSOME 20 OPEN READING FRAME 72	PEO [17]
**A. 2. Defective assembly of RC enzyme complex assembly**			
*BCS1L* (2q35)	7	BCS1, S. CEREVISIAE, HOMOLOG-LIKE, Complex III	GRACILE [18]
*SCO1* (17p13.1)	6	CYTOCHROME OXIDASE- 1	Hepatic failure [19]
**A. 3. Defective translation factors for RC enzyme complex assembly**			
TRMU (22q13.31)	11	tRNA 5-METHYLAMINOMETHYL-2-THIOURIDYLATE METHYLTRANSFERASE	Hepatic failure [20]
*GFM1* (3q25.32)	20	MITOCHONDRIAL ELONGATION FACTOR G1	OXPHOS deficiency 1 [21]
*TUFM* (16q11.2)	10	Tu TRANSLATION ELONGATION FACTOR, MITOCHONDRIAL	OXPHOS deficiency 4 [22]
**B. Fatty Acid Oxidation (FAO) defect disorders**			
HADHA (2p23.3)	19	HYDROXYACYL-CoA DEHYDROGENASE/3-KETOACYL-CoA THIOLASE/ENOYL-CoA HYDRATASE, ALPHA SUBUNIT	HELLP, LCHAD Deficiency, MTP deficiency [23]
*HADHB* (2p23.3)	16	HYDROXYACYL-CoA DEHYDROGENASE/3-KETOACYL-CoA THIOLASE/ENOYL-CoA HYDRATASE, BETA SUBUNIT	MTP deficiency [23]
*CPT1A* (11q13.3)	20	CARNITINE PALMITOYLTRANSFERASE I, LIVER	CPT deficiency type IA [24]
*CPT2* (1p32.3)	5	CARNITINE PALMITOYLTRANSFERASE II	CPT deficiency type II [25]
*ACADM* (1p31.1)	14	ACYL-CoA DEHYDROGENASE, MEDIUM-CHAIN	MCAD deficiency [26]
*ACAD9* (3q21.3)	18	ACYL-CoA DEHYDROGENASE FAMILY, MEMBER 9	ACAD9 deficiency [27]

Before interpretation, the data were analyzed and annotated by means of a pipeline that was developed in-house. Briefly, the output data from the HiSeq 2000 were converted from a bcl file to a FastQ file by means of Illumina Consensus Assessment of Sequence and Variation software and mapped to the reference haploid human-genome sequence (hg19) with the use of the NexGENe 2.2.3 program (Softgenetics). Variant calls, which differed from the reference sequence, were obtained with NextGENe 2.2.3. Variant prioritization was based on allele frequency, pathogenicity program predictions and mutation database searches. Alamut HT 1.1.8 and in-house scripts annotated the variants. Variants in this database with a minor allele frequency of less than 1% according to exome sequencing project database (http://evs.gs.washington.edu/EVS), and within +/- 20 bp of the exon/intron boundary were retained. In addition, damaging mutations were examined by focusing on frameshift, start loss and nonsense changes, splice site mutations and missense changes with pathogenic scores as predicted by SIFT, POLYPHEN-2 and Grantham scores. Moreover, variants reported in the Human Gene Mutation Database were also prioritized. Post-filtering, promising candidate gene variants were confirmed by Sanger sequencing.

### Respiratory chain (RC) complex activity

Frozen liver tissue from subjects #3, #11, #12 and from control subjects without acute or chronic liver disease were subjected to assays as previously described [12]. Specifically, the activity of complex I (NADH dehydrogenase) was determined by detecting rotenone (4 μM)-sensitive NADH oxidation at 340 nm with the coenzyme Q analogue, 2, 3-dimethyl-5-methyl 6-n-decyl-1,4-benzomethyluinone (DB), as an electron acceptor [13,14]. The activity of complex II (succinate dehydrogenase) was analyzed by detecting the secondary reduction of 2, 6-dichlorophenolindophenol by ubiquinone-2 at 600 nm [13,14]. Complex III (cytochrome *bc1* complex) activity was determined by measuring the reduction of cytochrome *c* at 550 nm with reduced decylubiquinone [13,14]. Complex IV (cytochrome *c* oxidase) activity was measured by monitoring the oxidation of reduced cytochrome *c* at 550 nm [13,14]. All complex activities were normalized with Citrate synthase, which was quantified by measuring the reduction of 5, 5’-dithiobis-2-nitrobenzoic acid at 412 nm in the presence of acetyl-CoA and oxaloacetate.

mtDNA copy number was determined on hepatic DNA from subjects with suspected MDS and from age-matched controls by quantitative PCR with primers and probes against the mitochondrial genes *CytB* and *Cox2* and the nuclear gene *B2M*, as previously reported [15]. Briefly, the relative mtDNA copy number was measured by a quantitative real-time polymerase chain reaction (QPCR) using an Applied Biosystems 7900HT Sequence Detection System (Applied Biosystems, Foster City, CA). Reactions were performed using a PrefeCTa SYBR Green FastMix kit (Quanta Biosciences), and corrected by simultaneous measurement of the nuclear DNA. The primer sequences for the mitochondrial *CytB* gene were: forward primer, 5′- tcattattgcagccctagcag, reverse primer, 5′- gttgtttgatcccgtttcgt, The forward and reverse primers of COX2 were 5′- gatccctcccttaccatcaaa and 5′- gccgtagtcggtgtactcgt. The primer pair used for the amplification of the nuclear gene *B2M* was as follows: forward primer, 5′- ggcttgaggtccgtagttga -3′; reverse primer, 5′- tttgtagagaccaggcttcacc, respectively. After denaturation at 95°C for 300 seconds, DNA samples were treated at 95°C for 15 seconds, 58°C for 15 seconds, and 60°C for 60 seconds for 40 cycles. Standard deviations for the cycle of threshold (Ct) duplicates were ≤0.2.

### Western blot

Protein lysates from frozen liver tissue of subject ID#11 and from two controls without acute liver failure were prepared with tissue homogenization in RIPA buffer with protease inhibitors, sonication, and centrifugation as described previously [16]. Protein concentration was determined using a Bradford assay (Bio-Rad, Hercules, CA). Protein samples (80 μg/well) were resolved on a 4–12% Bis-Tris gel (Life Technologies, Grand Island, NY) and transferred to a 0.2 μm nitrocellulose membrane (Bio-Rad). Membranes were blocked with 10% Blotting Grade Blocker in TBST (Bio-Rad) for 1 hour at 25°C, incubated with primary antibody (Ab) overnight at 4°C, washed 3x in TBST, and incubated with HRP-conjugated secondary Ab (1:10,000 dilution) for 2 hours at 25°C. Blots were developed with enhanced chemiluminescence (ECL; Pierce Biotechnology, Rockford, IL) and read on a low-light digital camera (LAS-1000; Fujifilm Medical Systems USA, Stamford, CT). The membrane was incubated for 1 hour at 25°C with RestorePLUS (Thermo Scientific, Rockford, IL) and reprobed using anti-GAPDH as a loading control with the above technique. Primary Abs were purchased from Abcam (Cambridge, MA): rabbit anti-human p53R2 (#130321, 1:500 dilution, NP_001165948.1), mouse anti-GAPDH (#9484, 1:2000 dilution). Secondary Ab included: HRP-conjugated anti-rabbit IgG and anti-mouse IgG (Promega, Madison, WI).

### Histology

Archived paraffin-embedded liver tissue samples were subjected to routine staining procedures with hematoxylin and eosin, trichrome, reticulin, and periodic acid–Schiff diastase. Fresh frozen samples were stained with oil red-O (ORO) for lipids. Liver histology was analyzed by two pediatric pathologists (K.E.B. and R.S.), who were blinded to the molecular diagnosis, using previously reported features of mitochondrial hepatopathies [17]. For electron microscopy (EM), biopsy tissues were fixed in 3% buffered glutaraldehyde, and subjected to ultrathin sectioning and examination with a Zeiss 912 transmission electron microscope, as previously described [18].

### Statistical analysis

Unpaired t and Fisher exact tests were applied to test differences of continuous and categorical data, respectively, between groups for statistical significance.

## Results

### Analysis of cohort

22 of the 40 subjects with indeterminate ALF had blood molar L/P ratios recorded during the first 7 days of hospitalization. Stratification of these children by peak L/P ratios revealed that 14 of them presented with a borderline high (L/P = 20–25) or abnormal L/P ratio (L/P >25). These patients were younger and showed increased incidence of abnormal findings on head imaging compared with the 8 subjects with a normal L/P ratio <20 (**Table 3**). Frozen liver tissue for molecular and biochemical studies was available for 12 of the subjects with idiopathic ALF and elevated L/P ratio (**Table 4**). The majority were Caucasian (75%) and presented with ALF at a young age (median age of 30 months) with severe coagulopathy (median INR of 5.35), elevation of serum aminotransferases (median ALT of 1633 IU/L) and hyperammonemia (median NH3 of 132 μmol/L). The outcome was poor: five (41%) died within 6 months after presentation, including two after LTx.

**Table 3:** Clinical characteristics of subjects with indeterminate ALF stratified by blood molar L/P ratio at presentation.

	L/P≥20	L/P<20
Number of subjects (n)	14	8
Mean L/P ratio	25	13.8
Median age (months)	30	62
Abnormal head imaging/	6/8 (75%)	
Number of subjects with	Cerebral Edema (4)	
head imaging	Encephalomalacia (2)	0/6 (0%)
Survival with native liver	3/14 (21.4%)	3/8 (37.5%)

**Table 4:** Summary of clinical course for the 12 subjects enrolled into targeted NGS.

ID#	Age	Sex	Ethnicity	INR	ALT (IU/L)	cBili (mg/dL)	NH3 (μmol/L)	L/P	Outcome of ALF	Most recent follow up
1	10 m	F	Caucasian	22	2457	3.9	83	20	LTx HD13,	-
									Death HD82	
2	23 y	M	Caucasian	5.2	678	11.3	120	26	Death HD12	-
3	16 y	M	Hispanic	6.6	767	17.5	116	25	LTx HD3	Alive at age
										27. Normal
										Head CT
4	20 m	F	African	3.6	5622	0.6	NA	30	LTx HD2;	-
			American						Death HD3	
5	15 m	F	African	10.5	467	4.2	NA	22	LTx HD10	Alive at age
			American							12. Normal
										Head CT.
6	2 m	F	Caucasian	5.5	2273	0.4	201	33	Recovered	Alive at age.
										10
7	2 y	M	Caucasian	20	12671	1.9	551	20	LTx HD2	Alive at age 9
8	6 y	F	Caucasian	2.75	58	0.4	666	28	Death HD2	-
9	2 y	F	Caucasian	4	994	4.9	70	23	Recovered	Alive at age 5
10	4 y	M	Caucasian	2.6	2958	13.7	82	21	Recovered	Alive at age 5
11	3 y	M	Caucasian	2.9	9650	2.3	144	31	LTx HD2	Alive at age 7
12	1 d	M	Caucasian	>20	260	0.2	265	114	Death HD1	-

Age (m-months, y-years, d-days); Peak laboratory values within first 7 days of admission: NA- not available, cBili-conjugated Bilirubin, Outcome of ALF within 6 months after presentation, HD- hospital day

### Gene sequence analysis

Hepatic DNA of all 12 subjects was subjected to targeted NGS. The average depth coverage of target region was 1583X. Moreover, the percentage matching to the target region was >95% at 10X. Many sequence alterations were detected: variants with potential clinical significance were observed in five genes *RRM2B*, *ACAD9*, *DGUOK*, *POLG*, and *POLG2* (**Table 5**). A heterozygous insertion change c.210_211insC in *RRM2B* (NM_001172477.1) was found in two unrelated patients. It has not been reported in the ESP database, and was predicted to be pathogenic due to a frameshift that introduces a stop codon 11 amino acids downstream within the ribonucleotide reductase domain. The variant c.187G>T in *ACAD9* was predicted to prematurely truncate the protein after 62 amino acids. The c.941T>C variant in the same gene was predicted to change amino acid leucine for proline and has not been reported before. The missense variant c.509A>G (p.Q170R) in *DGUOK* has a global and Caucasian minor allele frequencies of 0.01 and 0.022, respectively, and was classified as variant of uncertain clinical significance (VUCS). We classified variant c.2492A>G in *POLG* and variant c.1158T>G (p.D386E) in *POLG2* as VUCS due to conflicting predictions from SIFT and the Grantham scale as well as literature searches.

**Table 5:** Sequence variants and mutations in genes associated with MDS and FAO.

ID#	Gene	Mutation	Amino Acid change	Grantham	SIFT	Interpretation
2	*POLG*	c.2492A>G (het)	p.Y831C	Radically	Tolerated	VUCS
				conservative		
3	*RRM2B*	c.210_211insC	NA	NA	NA	Pathogenic/
		(het)*				truncating
6	*DGUOK*	c.509A>G (het)	p.Q170R	Moderately	Deleterious	VUCS
				conservative		
11	*RRM2B*	c.210_211insC	NA	NA	NA	Pathogenic/
		(het)*				truncating
12	*ACAD9*	c. 941T>C	p.L314P	Moderately	Tolerated	VUCS
		(het)*		conservative		
		c.187G>T (het)	p.E63X	Truncating	NA	Pathogenic
	*POLG2*	c.1158T>G(het)	p.D386E	Moderately	Tolerated	VUCS
				conservative		

het- heterozygous

*novel variant- not previously reported

NA- not applicable

### Phenotype and sequence variant in a new ALF-associated gene

**Fig 1:**
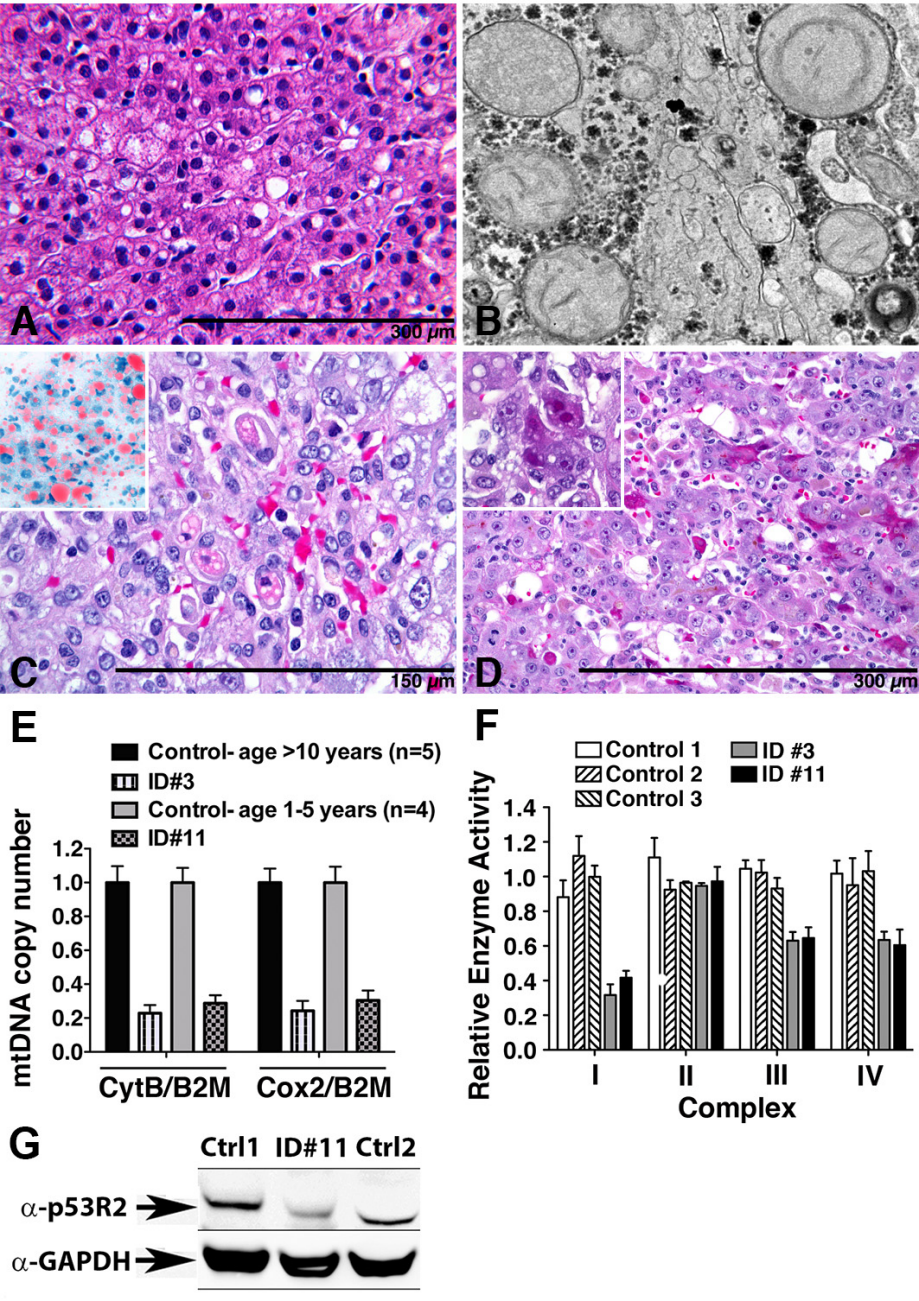
Liver histology, ultrastructure, mtDNA copy number and respiratory complex activity of subjects with heterozygous mutations in *RRM2B*. **(A)** Liver biopsy following the second episode of ALF in subject ID#11 showed mild steatosis. H&E stain. **(B)** Mitochondria at that time were normal in size with pale matrix and subtle haphazard arrangement of cristae. **(C** and **D)** Random, non-zonal macro- and microvesicular steatosis was prevalent in H&E- stained sections from the explanted livers of both subjects, confirmed with Oil red O in a frozen section (**C, insert).** Both explanted livers contained unusual necrotic/apoptotic hepatocytes with distorted contours, granular eosinophilic cytoplasm, and fat droplets. (**E**) Copy numbers of mtDNA in liver tissues from age-matched control subjects without ALF and from subjects #3 and #11 were determined by qPCR for the mitochondrial genes *CytB* and *Cox2*, normalized to the nuclear gene *B2M*. (**F**) Respiratory chain complex activities were simultaneously determined on frozen samples from explanted livers of ID#3 and ID#11 and from liver tissue of control subjects without ALF. (**G**)Total protein extracts from liver tissues from ID#11and from two age-matched controls without ALF were analyzed by SDS-PAGE and immunoblotting against p53R2, the 39kDa gene product of *RRM2B*.

Here, we report a heterozygous frameshift mutation in *RRM2B* in two subjects with ALF. Subject #3, a 16-year-old male from Puerto Rico, presented with three weeks history of malaise and fatigue, and underwent LTx on hospital day 2 for progressive coagulopathy. No evidence of mitochondrial multi-system disorder was recorded during follow-up of 11 years. Subject #11 presented with recurrent ALF. The first episode at age 20 months was associated with profound hypoglycemia and lactic acidosis; a second, 3 months later followed a febrile illness. The patient recovered spontaneously from both episodes, but then underwent LTx during the 3^rd^ episode because of progressive encephalopathy. Neurocognitive and motor development remained normal during four years of follow-up. Subject #11 had undergone a liver biopsy between the first two episodes of ALF, which revealed mild steatosis and subtle reactive changes in hepatocytes (**Fig 1A**). EM at this time showed minor abnormalities within many mitochondria (**Fig 1B**). Macro- and microvesicular steatosis, affecting approximately 65 and 80% of hepatocytes in subject #11 and about 40 and 5% of hepatocytes in subject #3, suggested impaired energy metabolism (**Fig 1C and 1D**). A striking change was the presence of individual apoptotic hepatocytes with granular oncocytic cytoplasm and lipid droplets (**Fig 1C and 1D**). For both subjects, hepatic DNA copy number for the mitochondrial genes *CytB* and *Cox2* were reduced to <30% compared with copy numbers in livers of age matched control subjects (**Fig 1E**). RC complex activity assays performed on frozen samples from explanted livers of both subjects showed significant reduction in activity of the mtDNA dependent complexes I, III and IV with most prominent reduction in complex I to 39% and 42% in subject #3 and #11, respectively, while the activity of complex II, encoded entirely by nuclear DNA, was preserved **(Fig 1F**). Immunoblotting of liver protein extract from subject #11 showed reduced expression of p53R2, a 39kDA protein encoded by *RRM2B*, compared with controls (**Fig 1G**). Severe global mtDNA depletion and reduced activity in several mtDNA dependent RC complexes, as shown for subjects #3 and #11, is typically found in ALF secondary to MDS.

### Liver phenotype of subjects with sequence variants in genes previously linked to ALF

**Fig 2:**
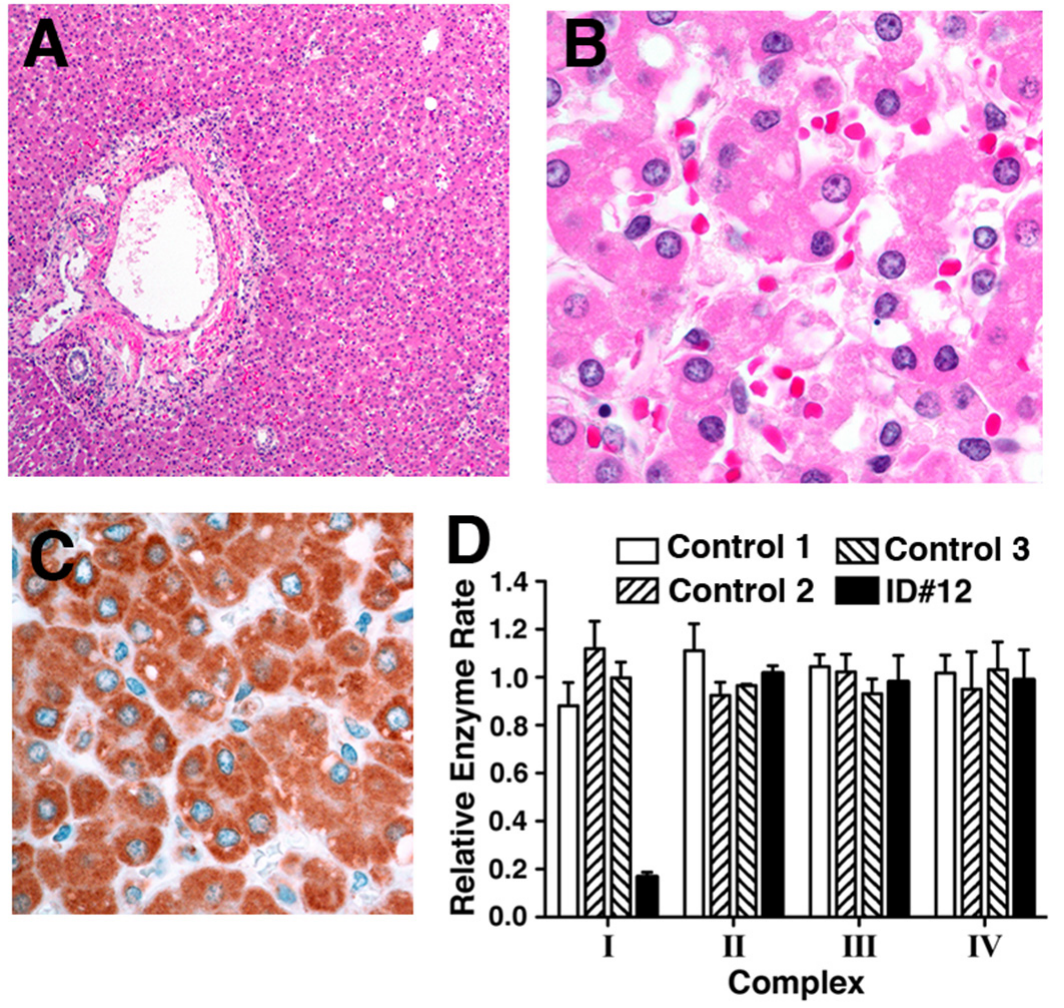
Isolated complex 1 deficiency in an infant with compound heterozygous mutations in *ACAD9*. Subject #12 harboring the variant L314P and the mutation E63X in *ACAD9* succumbed to ALF and multi-organ failure within the first 24 hours of life. Post-mortem, liver architecture was preserved without significant collapse or inflammation and with minimal steatosis (H&E, **A**). However, abnormally granular hepatocytes (H&E, **B**) and mitochondrial hyperplasia detected by immunohistochemistry against mitochondrial antigens (**C**) suggest a mitochondrial hepatopathy. Respiratory chain complex assay demonstrates reduced activity of complex 1 in frozen liver, compared to liver from controls without ALF (**D**).

Variants in *ACAD9*, encoding the mitochondrial enzyme acyl-CoA 9 dehydrogenase, were detected in subject #12, who developed liver and heart failure soon after birth. Autopsy showed preserved liver architecture with diffuse granular cytoplasmic changes (oncocyte like) in hepatocytes, and without significant collapse, or inflammation; steatosis was rare (**Fig 2A and 2B**). Abnormally granular hepatocytes in H&E stained liver sections (**Fig 2B**), and immunohistochemistry against mitochondrial antigens suggested mitochondrial proliferation (**Fig 2C**). RC activity on liver tissue showed reduction of activity for complex I to 17%, whereas the activities for complexes II, III, and IV ranged between 98 and 101% of normal controls (**Fig 2D**). The copy number for mtDNA encoding CytB and Cox2 was only mildly reduced to 67% and 63%, respectively, compared to 4 normal subjects <1 year (data not shown), indicating that ACAD9 deficiency caused complex I dysfunction rather than global mtDNA depletion. Although it is difficult in this case to determine the degree by which the liver dysfunction may be secondary to ischemia from circulatory collapse and cardiomyopathy, the severity of coagulopathy (INR>20), the liver histological and ultrastructural changes and the greatly reduced activity of complex I in the liver suggest that hepatocyte dysfunction linked to ACAD9 deficiency is the primary cause for ALF in this subject with multi-organ failure.

**Fig 3:**
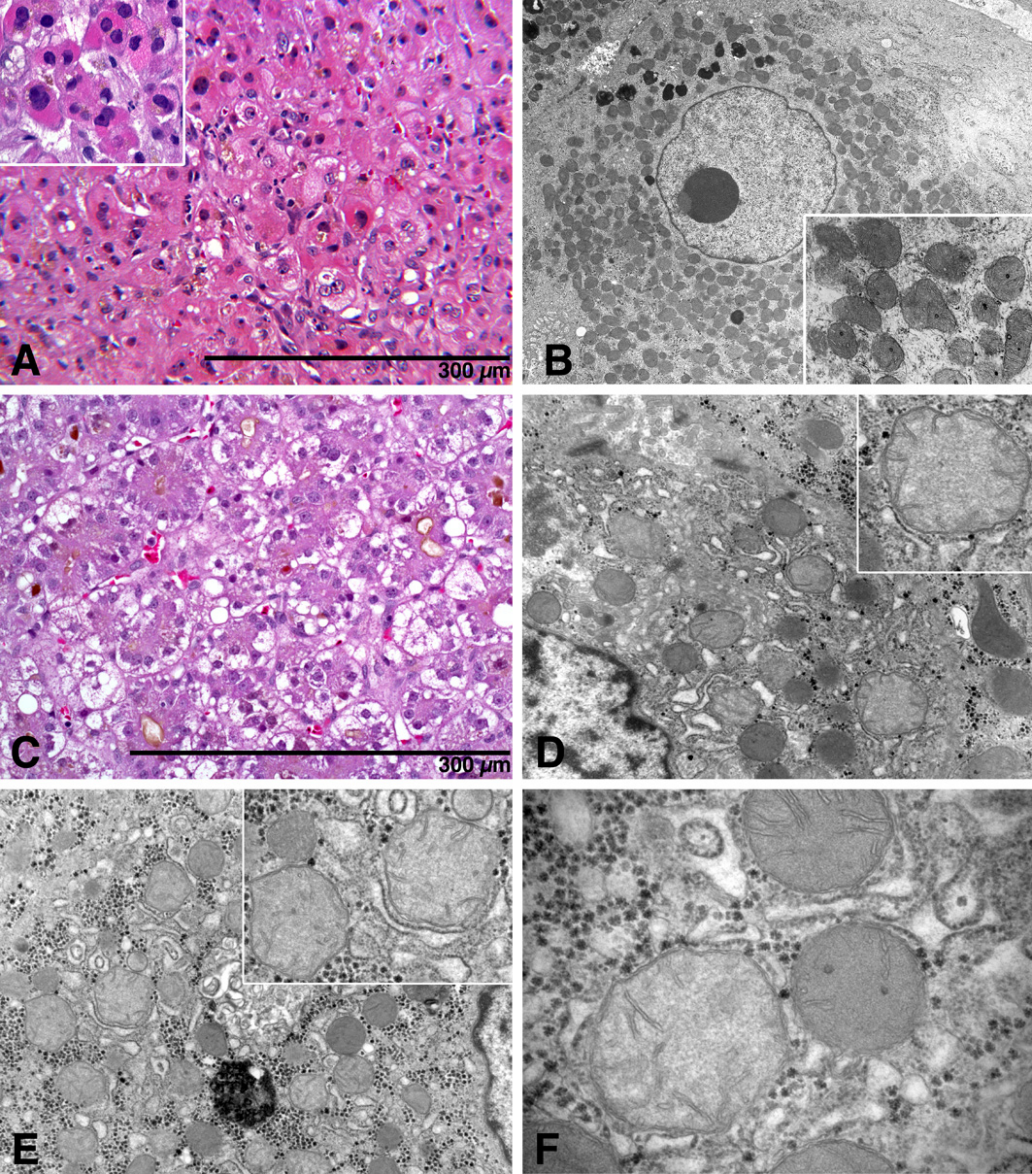
Heterozygous variants in *POLG* and *DGUOK* may predispose to drug-induced ALF with a histological phenotype of impaired OXPHOS. Subject # 2 with Y831C in *POLG* developed fatal ALF following exposure to dantrolene. (**A**) Hepatocytes at time of diagnosis revealed mild random steatosis, and prominent oncocytic changes in isolated and clustered hepatocytes (**insert**). H&E stain. (**B**). Concurrent ultrastructure study demonstrated numerical excess of mitochondria accompanied by mild pleomorphism (**insert**). Subject #6 with Q170R in *DGUOK* developed ALF following exposure to acetaminophen. (**C**) Liver showed lobular cholestasis, prominent pseudoacinar transformation, hepatocyte swelling, and moderate non-zonal macro- and microvesicular steatosis. (**D-F**) Concurrent ultrastructure study revealed several populations of mitochondria in many hepatocytes of which three are illustrated. Intermixed with normal mitochondria were numerous mitochondria that displayed “Reye-syndrome”- like changes, including ameboid shape and watery degenerative matrix.

Two subjects with heterozygous VUCS in *POLG* (ID #2) and *DGUOK* (ID#6) developed ALF following drug exposure. Heterozygous common variants in these two genes have been linked to drug induced liver injury before [1, 2]. However, these prior studies did not have access to liver tissue to discern liver histology and specifically ultrastructural integrity of mitochondria. In our case series, subject #2, a 23-year-old male sustained two cerebrovascular accidents after an astrocytoma was resected at age 18 months followed by radiation therapy. He had been treated with dantrolene (300 mg/day) for spastic quadriplegia for 5 years prior to presentation with ALF. Liver biopsy revealed mild to moderate macro- and microvesicular steatosis, frank oncocytic changes in hepatocytes and mild inflammation (**Fig 3A**). EM revealed that many hepatocytes contained increased numbers of mitochondria with mild pleomorphism **(Fig 3B**). The explanted liver showed similar histology. Subject #6, a 2-month-old, full-term infant who was treated with acetaminophen for fever and congestion for 2 weeks prior to presentation with ALF was found to have an elevated APAP level of 52 μg/ml at diagnosis. A percutaneous liver biopsy failed to demonstrate typical centrilobular necrosis of APAP toxicity, but instead showed lobular cholestasis with prominent pseudoacinar transformation of hepatocytes, and non-uniform, mild macro and microvesicular steatosis (**Fig 3C**). Liver ultrastructure showed a subpopulation of mitochondria with decreased matrix density and mild ameboid pleomorphism (**Fig 3D–3F**). Although the mtDNA copy numbers for *CytB* and *Cox2* were >92% of age-matched controls in both subjects (data not shown), steatosis and ultrastructural changes in mitochondrial morphology suggested impaired energy metabolism.

## Discussion

We determined in a retrospective, single center cohort of children with ALF that elevated a borderline high (L/P = 20–25) or abnormal L/P ratio (L/P >25) at diagnosis is associated with young age at presentation suggesting that an occult metabolic disease may be the predisposing condition, which have previously been reported in mitochondrial disorders [5,6,10]. In a proof of principle study, hepatic DNA from 12 subjects with ALF of indeterminate etiology and elevated L/P ratio was subjected to targeted NGS and pathogenic variants associated with MDS or FAO and VUCS were detected in five. We found an association between heterozygous deleterious mutations in *RRM2B* and liver tissue evidence of global mtDNA depletion. A molecular diagnosis of *ACAD9* deficiency was established for an infant with a clinical phenotype of multi-organ failure as seen in hepatocerebral MDS. Heterozygous variants in the genes *POLG* and *DGUOK*, previously shown to be implicated in hepatocerebral MDS when affected by bi-allelic mutations, were associated with ALF following drug exposure [19,20]. Hepatic steatosis and oncocytosis are commonly associated with mutations in MDS associated genes.

We detected a novel frameshift mutation predicted to prematurely truncate the p53R2 protein by introducing a stop codon 11 amino acids downstream in the ribonucleotide reductase domain of *RRM2B* in two unrelated subjects with molecular evidence of MDS, including reduced mtDNA copy number and depressed activity for the mtDNA dependent RC complexes. We conclude that the deleterious mutation in *RRM2B* acted in autosomal-dominant fashion based on reduced (but not absent) expression of the encoded protein p53R2. Ribonucleotide reductase small subunit inducible by the tumor suppressor p53 is essential for dNTPs synthesis required for DNA repair and mtDNA synthesis in non-proliferating cells. Bi-allelic mutations were reported to cause MDS type-8A characterized by neonatal hypotonia, lactic acidosis, neurologic deterioration, and renal tubular defects [21]. Dominantly inherited heterozygous mutations in the same isoform of *RRM2B* were associated with a myopathic phenotype with onset in adulthood [22]. Liver disease, including ALF, has not been reported in any of the cases of recessively or dominantly inherited *RRM2B*-associated neuromuscular disorder. Importantly, the clinical outcome was excellent in both subjects of our case series, without obvious neuromuscular disease at 11 and 5 years following LTx. This contrasts with the experience in ALF due to hepatocerebral MDS from bi-allelic mutations in *POLG*, *DGUOK* or *MPV17* commonly manifesting as multi-system disorders before or shortly after onset of liver disease [23]. Limitations of this retrospective study include the lack of DNA samples of parents and family members to interrogate inheritance of the variants. Whether the disease phenotype of liver restricted MDS due to heterozygous mutations in *RRM2B* can be explained on the basis of autosomal dominant inheritance, or originated from a de-novo mutation remains elusive. Mechanisms of liver injury in p53R2 deficiency may be linked its primary function in supplying dNTP for mtDNA replication and DNA repair, but also to its role in controlling key mitochondrial functions, like ATP synthesis, cytochrome c oxidase activity and membrane potential maintenance [24,25]. Large scale proteomics efforts have recently detected eight isoforms of RRM2B and began to further our understanding of the expression patterns [26]. Noteworthy, data are limited on the isoform NM_001172477.1 of RRM2B, in which our heterozygous variant was found in and for which we show reduced expression by Western blotting. It is possible that this is a liver specific isoform.

Our finding that bi-allelic, c. 941T>C and c.187G>T, mutations in *ACAD9* may present with lactic acidosis and severe coagulopathy phenocopying severe MDS highlights the importance to consider NGS early in the diagnostic evaluation of infants with ALF. *ACAD9* has ascribed functions in FAO and in mitochondrial biogenesis, based on its dehydrogenase activity for very long chain fatty acids as well as its role in the assembly of the RC complex 1, respectively [27,28]. Recurrent ALF with severe elevation of serum aminotransferases and hypoglycemia with onset in infancy has been reported for one patient [29]. Although it is beyond the scope of this manuscript to elucidate to what degree ACAD9 deficiency affected other organs in this patient with multi-organ failure, our data suggest it be included in the differential diagnosis for ALF in infants. Unlike subjects #3 and #11 with *RRM2B* mutations and typical findings of mtDNA depletion induced hepatopathy, the liver of ID#12 showed near normal mtDNA copy number and no steatosis. Given the distinct changes in RC complex activity in liver tissue, which had previously been ascribed to bi-allelic *ACAD9* mutations, our studies strongly suggests that the variant c.941T>C should be considered a pathogenic mutation and that the two variants c.187G>T and c.941T>C act in trans, despite lack of confirmation of inheritance using parental DNA. The variant c.187G>T in ACAD9 has been reported to be disease-causing in a compound heterozygous state [27]. Interestingly, c.1158T>G (p.D386E) in *POLG2*, found in ID#12, was previously detected in an infant with ALF, however, the in vitro activity of the recombinant protein did not change and may or may not be a contributing factor to the clinical features of ID#12 [30]. Importantly, riboflavin was found to act as a chaperone in increasing the protein levels of mutated ACAD9, to increase complex 1 activity in fibroblasts from patients with ACAD9 deficiency, and to alleviate symptoms in children with ACAD9 associated progressive encephalomyopathy [31–33]. Response may be mutation specific since a recent report failed to demonstrate restoration of complex 1 activity in fibroblasts from a patients with bialleleic mutations in *ACAD9* [34].

In two subjects, we detected VUCS in *POLG* and *DGUOK* which may have predisposed to fulminant drug-induced liver injury. Importantly, we report a spectrum of histomorphological changes associated with impaired energy metabolism, including micro-and macrovesicular steatosis in both cases, and ultrastructural changes in the mitochondria, including ameboid pleomorphism in subject #6. The latter changes in mitochondria were similar to those described in Reye syndrome linked to salicylate exposure [35]. Although the evidence for a causative role of the variants is circumstantial given the lack of functional data for the variants, and the possibility that the mitochondrial changes are reactive, our report contributes to an evolving understanding of the role of heterozygous mutations in genes associated with mitochondrial biogenesis and function as risk factors for drug induced liver disease. Mutations in *POLG* have not been linked to well described hepatic adverse reactions of dantrolene, but are well-known susceptibility factors for sodium valproate-induced liver injury, even as common variants in the heterozygous state [36,37]. Furthermore, the variant Q170R in *DGUOK* in heterozygous state has been detected in a cohort of 45 subjects with ALF of which one developed liver failure following exposure to isoniazid [38,39].To what extent this variant contributed to the APAP induced ALF in subjects #6 requires further functional studies.

In summary, mutations in genes associated with MDS or FAO defects are important susceptibility factors in a proportion of children with ALF of indeterminate etiology and clinical (blood L/P ratio > 20) or morphological features of impaired energy metabolism (hepatic steatosis, oncocytic changes, or ultrastructural mitochondriopathy). Early diagnosis by targeted NGS may be of critical importance to guide clinical intervention with a reduction of turnaround time by rapid next generation sequencing and bioinformatic analysis solutions, including selecting the appropriate candidates for LTx. and defining those who are at risk for recurrence due to an inherited metabolic defect. Our results in a retrospective cohort suggest that the impact of NGS on diagnosis and treatment of pediatric patients with idiopathic ALF and elevated plasma molar L/P ratio should be studied in prospective and multi-center fashion using an infrastructure similar to the PALF research consortium currently available in the US and Canada.

## Abbreviations

ALF: acute liver failure
L/P: lactate/pyruvate
MDS: mitochondrial DNA depletion syndrome
NGS: next-generation sequencing
mtDNA: mitochondrial DNA
OXPHOS: oxidative phosphorylation
LTx: Liver transplantation
EM: electron microscopy
RC: respiratory chain
CGH: Comparative genomic hybridization
VUCS: variant of unknown clinical significance

## References

[1] Sundaram SS, Alonso EM, Narkewicz MR, Zhang S, Squires RH, Pediatric Acute Liver Failure Study Group. Characterization and outcomes of young infants with acute liver failure. J Pediatr. 2011;159: 813–818.e1. 10.1016/j.jpeds.2011.04.016 21621221 PMC3177978

[2] Brett A, Pinto C, Carvalho L, Garcia P, Diogo L, Gonçalves I. Acute liver failure in under two year-olds—are there markers of metabolic disease on admission? Ann Hepatol. 2013;12: 791–796. 24018497

[3] Copeland WC. Defects in mitochondrial DNA replication and human disease. Crit Rev Biochem Mol Biol. 2012;47: 64–74. 10.3109/10409238.2011.632763 22176657 PMC3244805

[4] Sarzi E, Bourdon A, Chrétien D, Zarhrate M, Corcos J, Slama A, et al Mitochondrial DNA depletion is a prevalent cause of multiple respiratory chain deficiency in childhood. J Pediatr. 2007;150: 531–534, 534.e1–6. 10.1016/j.jpeds.2007.01.044 17452231

[5] Fellman V, Kotarsky H. Mitochondrial hepatopathies in the newborn period. Semin Fetal Neonatal Med. 2011;16: 222–228. 10.1016/j.siny.2011.05.002 21680270

[6] Honzik T, Tesarova M, Magner M, Mayr J, Jesina P, Vesela K, et al Neonatal onset of mitochondrial disorders in 129 patients: clinical and laboratory characteristics and a new approach to diagnosis. J Inherit Metab Dis. 2012;35: 749–759. 10.1007/s10545-011-9440-3 22231385

[7] Calvo SE, Compton AG, Hershman SG, Lim SC, Lieber DS, Tucker EJ, et al Molecular diagnosis of infantile mitochondrial disease with targeted next-generation sequencing. Sci Transl Med. 2012;4: 118ra10 10.1126/scitranslmed.3003310 22277967 PMC3523805

[8] Vilarinho S, Choi M, Jain D, Malhotra A, Kulkarni S, Pashankar D, et al Individual exome analysis in diagnosis and management of paediatric liver failure of indeterminate aetiology. J Hepatol. 2014;61: 1056–1063. 10.1016/j.jhep.2014.06.038 25016221 PMC4203706

[9] Molleston JP, Sokol RJ, Karnsakul W, Miethke A, Horslen S, Magee JC, et al Evaluation of the child with suspected mitochondrial liver disease. J Pediatr Gastroenterol Nutr. 2013;57: 269–276. 10.1097/MPG.0b013e31829ef67a 23783016 PMC3810178

[10] Sokal EM, Sokol R, Cormier V, Lacaille F, McKiernan P, Van Spronsen FJ, et al Liver transplantation in mitochondrial respiratory chain disorders. Eur J Pediatr. 1999;158 Suppl 2: S81–84. 10603105 10.1007/pl00014328

[11] Liu Z-J, Li H-F, Tan G-H, Tao Q-Q, Ni W, Cheng X-W, et al Identify mutation in amyotrophic lateral sclerosis cases using HaloPlex target enrichment system. Neurobiol Aging. 2014;35: 2881.e11–15. 10.1016/j.neurobiolaging.2014.07.003 25109764

[12] Tang S, Le PK, Tse S, Wallace DC, Huang T. Heterozygous mutation of Opa1 in Drosophila shortens lifespan mediated through increased reactive oxygen species production. PloS One. 2009;4: e4492 10.1371/journal.pone.0004492 19221591 PMC2637430

[13] Barrientos A. In vivo and in organello assessment of OXPHOS activities. Methods San Diego Calif. 2002;26: 307–316. 10.1016/S1046-2023(02)00036-1 12054921

[14] Trounce IA, Kim YL, Jun AS, Wallace DC. Assessment of mitochondrial oxidative phosphorylation in patient muscle biopsies, lymphoblasts, and transmitochondrial cell lines. Methods Enzymol. 1996;264: 484–509. 8965721 10.1016/s0076-6879(96)64044-0

[15] Wong A, Cortopassi G. Reproducible quantitative PCR of mitochondrial and nuclear DNA copy number using the LightCycler. Methods Mol Biol Clifton NJ. 2002;197: 129–137. 10.1385/1-59259-284-8:129 12013791

[16] Rudolph JA, Pratt J, Mourya R, Steinbrecher KA, Cohen MB. Novel mechanism of cyclic AMP mediated extracellular signal regulated kinase activation in an intestinal cell line. Cell Signal. 2007;19: 1221–1228. 10.1016/j.cellsig.2007.01.002 17317103

[17] Ducluzeau PH, Lachaux A, Bouvier R, Streichenberger N, Stepien G, Mousson B. Depletion of mitochondrial DNA associated with infantile cholestasis and progressive liver fibrosis. J Hepatol. 1999;30: 149–155. 9927162 10.1016/s0168-8278(99)80019-1

[18] Wong L-JC, Brunetti-Pierri N, Zhang Q, Yazigi N, Bove KE, Dahms BB, et al Mutations in the MPV17 gene are responsible for rapidly progressive liver failure in infancy. Hepatol Baltim Md. 2007;46: 1218–1227. 10.1002/hep.21799 17694548

[19] Mancuso M, Filosto M, Oh SJ, DiMauro S. A novel polymerase gamma mutation in a family with ophthalmoplegia, neuropathy, and Parkinsonism. Arch Neurol. 2004;61: 1777–1779. 10.1001/archneur.61.11.1777 15534189

[20] Tang S, Wang J, Lee N-C, Milone M, Halberg MC, Schmitt ES, et al Mitochondrial DNA polymerase gamma mutations: an ever expanding molecular and clinical spectrum. J Med Genet. 2011;48: 669–681. 10.1136/jmedgenet-2011-100222 21880868

[21] Bourdon A, Minai L, Serre V, Jais J-P, Sarzi E, Aubert S, et al Mutation of RRM2B, encoding p53-controlled ribonucleotide reductase (p53R2), causes severe mitochondrial DNA depletion. Nat Genet. 2007;39: 776–780. 10.1038/ng2040 17486094

[22] Pitceathly RDS, Smith C, Fratter C, Alston CL, He L, Craig K, et al Adults with RRM2B-related mitochondrial disease have distinct clinical and molecular characteristics. Brain J Neurol. 2012;135: 3392–3403. 10.1093/brain/aws231 PMC3501970 23107649

[23] Al-Hussaini A, Faqeih E, El-Hattab AW, Alfadhel M, Asery A, Alsaleem B, et al Clinical and molecular characteristics of mitochondrial DNA depletion syndrome associated with neonatal cholestasis and liver failure. J Pediatr. 2014;164: 553–559.e1–2. 10.1016/j.jpeds.2013.10.082 24321534

[24] Guittet O, Tebbi A, Cottet M-H, Vésin F, Lepoivre M. Upregulation of the p53R2 ribonucleotide reductase subunit by nitric oxide. Nitric Oxide Biol Chem Off J Nitric Oxide Soc. 2008;19: 84–94. 10.1016/j.niox.2008.04.011 18474260

[25] Wang X, Liu X, Xue L, Zhang K, Kuo M-L, Hu S, et al Ribonucleotide reductase subunit p53R2 regulates mitochondria homeostasis and function in KB and PC-3 cancer cells. Biochem Biophys Res Commun. 2011;410: 102–107. 10.1016/j.bbrc.2011.05.114 21640705 PMC3180957

[26] Wilhelm M, Schlegl J, Hahne H, Moghaddas Gholami A, Lieberenz M, Savitski MM, et al Mass-spectrometry-based draft of the human proteome. Nature. 2014;509: 582–587. 10.1038/nature13319 24870543

[27] Nouws J, Nijtmans L, Houten SM, van den Brand M, Huynen M, Venselaar H, et al Acyl-CoA dehydrogenase 9 is required for the biogenesis of oxidative phosphorylation complex I. Cell Metab. 2010;12: 283–294. 10.1016/j.cmet.2010.08.002 20816094

[28] Zhang J, Zhang W, Zou D, Chen G, Wan T, Zhang M, et al Cloning and functional characterization of ACAD-9, a novel member of human acyl-CoA dehydrogenase family. Biochem Biophys Res Commun. 2002;297: 1033–1042. 12359260 10.1016/s0006-291x(02)02336-7

[29] He M, Rutledge SL, Kelly DR, Palmer CA, Murdoch G, Majumder N, et al A new genetic disorder in mitochondrial fatty acid beta-oxidation: ACAD9 deficiency. Am J Hum Genet. 2007;81: 87–103. 10.1086/519219 17564966 PMC1950923

[30] Young MJ, Longley MJ, Li F-Y, Kasiviswanathan R, Wong L-J, Copeland WC. Biochemical analysis of human POLG2 variants associated with mitochondrial disease. Hum Mol Genet. 2011;20: 3052–3066. 10.1093/hmg/ddr209 21555342 PMC3131046

[31] Lucas TG, Henriques BJ, Rodrigues JV, Bross P, Gregersen N, Gomes CM. Cofactors and metabolites as potential stabilizers of mitochondrial acyl-CoA dehydrogenases. Biochim Biophys Acta. 2011;1812: 1658–1663. 10.1016/j.bbadis.2011.09.009 21968293

[32] Haack TB, Danhauser K, Haberberger B, Hoser J, Strecker V, Boehm D, et al Exome sequencing identifies ACAD9 mutations as a cause of complex I deficiency. Nat Genet. 2010;42: 1131–1134. 10.1038/ng.706 21057504

[33] Gerards M, van den Bosch BJC, Danhauser K, Serre V, van Weeghel M, Wanders RJA, et al Riboflavin-responsive oxidative phosphorylation complex I deficiency caused by defective ACAD9: new function for an old gene. Brain J Neurol. 2011;134: 210–219. 10.1093/brain/awq273 20929961

[34] Nouws J, Wibrand F, van den Brand M, Venselaar H, Duno M, Lund AM, et al A Patient with Complex I Deficiency Caused by a Novel ACAD9 Mutation Not Responding to Riboflavin Treatment. JIMD Rep. 2014;12: 37–45. 10.1007/8904_2013_242 23996478 PMC3897792

[35] Partin JS, Daugherty CC, McAdams AJ, Partin JC, Schubert WK. A comparison of liver ultrastructure in salicylate intoxication and Reye’s syndrome. Hepatol Baltim Md. 1984;4: 687–690. 10.1002/hep.1840040421 6745858

[36] Chan CH. Dantrolene sodium and hepatic injury. Neurology. 1990;40: 1427–1432. 2392230 10.1212/wnl.40.9.1427

[37] Nguyen KV, Sharief FS, Chan SSL, Copeland WC, Naviaux RK. Molecular diagnosis of Alpers syndrome. J Hepatol. 2006;45: 108–116. 10.1016/j.jhep.2005.12.026 16545482

[38] Freisinger P, Fütterer N, Lankes E, Gempel K, Berger TM, Spalinger J, et al Hepatocerebral mitochondrial DNA depletion syndrome caused by deoxyguanosine kinase (DGUOK) mutations. Arch Neurol. 2006;63: 1129–1134. 10.1001/archneur.63.8.1129 16908739

[39] Helbling D, Buchaklian A, Wang J, Wong L-J, Dimmock D. Reduced mitochondrial DNA content and heterozygous nuclear gene mutations in patients with acute liver failure. J Pediatr Gastroenterol Nutr. 2013;57: 438–443. 10.1097/MPG.0b013e31829ef4b4 23783014 PMC4966813

